# Cases from Practice

**Published:** 1882-09

**Authors:** J. B. Gregg Custis

**Affiliations:** Washington, D. C.; Bureau Clinical Medicine, I. H. A.


### CASES FROM PRACTICE.

J. B. Gregg Custis, M. D., Washington, D. C.

(*Bureau Clinical Medicine, I. H. A.*)

Aug. 6th, 1881. I was called to E. C., female, æt. two years and nine months, of light complexion and hair, with large head and protruding forehead. Her father died of phthisis pulmonalis, two months before her birth. Three days before being called she had been exposed to the sun through the carelessness of her nurse. She was attacked with frequent vomiting of small quantities, especially after drinking water, for which she was eager, accompanied by diarrhœa of small, frequent, watery stools, with great prostration. I gave Ars. 50M.

Aug. 8th. Vomiting and diarrhœa checked; still greatly prostrated and inclined to sleep, but would start up sometimes with a cry; face alternately red and pale; pupils dilated; pulse full and rolling under the finger; would eat nothing and showed little desire to drink. Gave Bell.$^{200}$, and, in view of family history, gave a guarded prognosis.

Aug. 9th. No stool during the night, but three forcibly-expelled yellow stools during the morning, accompanied by colic, which was relieved by the stool; very little urine passed. Gave Gambogia$^{200}$, to be repeated after each stool.

Aug. 10th. No stool after second dose, but no urine passed for fifteen hours, excepting a spoonful, which was nearly black; pulse, about seventy, with marked loss of strength on the left side of the body. Gave Hellebore$^{200}$, and made doubtful prognosis with diagnosis of tubercular meningitis.

Aug. 11th. A slight quantity of urine passed; no movement of the bowels; less rolling of the head, but a sinking in of the abdomen; left side no worse; considerable sweat on the occiput; no appetite, but had been coaxed to take a small cup of milk.

Though there was slight improvement, the appearance of the child called so loudly for Calc. carb., that I changed the remedy.

Aug. 12th. A slight improvement, a little more nourishment taken; more urine passed, but no stool since the 8th. Continued the remedy.

Aug. 13th. Weather very sultry, and our little patient weaker, though in other respects brighter, having taken more nourishment, passed more water, and taken some notice of her doll.

Continued the same remedy, and advised the mother to leave the city immediately for the North, stopping in Philadelphia long enough to consult Dr. C. C. Smith.

She left at night and saw Dr. Smith in the morning. He confirmed my diagnosis and, on account of her father's history, gave a dose of Tuberculinum$^{mm}$, upon which she continued to improve for four days, when, on account of crossness and feverishness after every nap and the appearance of red sand in the urine, he gave a dose of Lyc.$^{cm}$

This was all the medicine she received until Oct. 8th, when she returned in apparently good health, though entirely changed in appearance and disposition.

Some may be unwilling to admit the diagnosis of our case, though none will question the congestion of the brain.

We do not hesitate to say that a large percentage of the fatal cases ascribed to enteric disease, have their origin in the brain; in summer the first stage being congestion of the brain, caused by heat, the last symptoms being due to ænemia.

Cases of diarrhœa from irritation of the stomach or bowels, if not cured, will end in marasmus, while those having their origin in the brain will, if fatal, end in convulsions in seven days, or in some multiple of seven.

We would ask that some of our older members would mark out the relation of the brain to the bowel symptoms of our remedies.

We based the diagnosis in the above case on the history, the following constipation, the suppression of the urine, the sinking in of the abdomen and the approaching paralysis.

Case second.—Sept. 23d, 1881. Called to see C. H., male, æt. eight years, dark complexion and hair, large head and very small limbs.

Found him with his head thrown back, right leg drawn up, left perfectly straight; seemed sore and would cry upon being moved; lumbar vertebræ sensitive to the touch; temperature of the body normal; could stand on the right foot, but had no power over the left leg and but little strength in the left hand.

He would eat nothing but meat, nor drink anything but soup made of meat. He had been in this condition for several days, but having no fever his parents thought it would pass off.

On account of his desire for meat, and the large, crumbling, infrequent stools, I gave Mag. carb.$^{50m}$, upon which he commenced and continued to improve for some days, being able to sit up and play and move about, though he would drag the left leg.

His diet also became more extended, though the tenderness of the skin continued, on account of which I gave Gettysburg salt$^{cm}$.

He had no other medicine excepting an intercurrent dose of Nat. mur., given on account of his aversion to bread, and longing for salt.

In a short time his recovery was complete.

Case third.—E. G., female, æt. nine months; large head, light hair and complexion. Her father died of phthisis.

She had long-continued crying spells, accompanied by a desire to pinch everybody and everything.

The attacks would last for hours, during which time it was impossible to induce sleep. This case was cured by Coffea$^{10m}$.

The symptom of pinching everything was cured in another child by the same remedy. This latter also developed the inordinate appetite for meat, which was corrected by Mag. carb.$^{50m}$.

I have frequently noticed this symptom in children of tuberculous parentage, and in many cases Mag. carb. has been a constitutional remedy for their stomach disorders.

Though not within the province of our bureau, there is another cured symptom to which I beg leave to call your attention, in

Case fourth.—E. N., male, æt. fifty years, severe neuralgia of the stomach. So diagnosed by two old school physicians, who had treated him for two months, during which time his attacks had greatly increased in severity, and reduced him twenty pounds in flesh. Attacks irregular, coming on without special cause; pain in the pit of the stomach, with distressing burning and soreness. Wanted the clothing all loose, and to bend back, with great restlessness and some jaundice.

After trying several remedies which I thought to be indicated, on one occasion, when he said that he thought that he was about to have another attack, I noticed him scratching the palms of his hands. Calling his attention to it, he said that the attacks were generally preceded by this symptom. Having read, in The Homœopathic Physician, of an itching eruption in the palms of the hand being cured by Dr. Lippe with Ranunculus bulb, I gave him a few powders of the 200th, and he has not had an attack from that day, now six months past.

Since then I have cured a case of jaundice, with itching of the body, especially of the palms of the hands, with the same remedy

